# Decreased Taxon-Specific IgA Response in Relation to the Changes of Gut Microbiota Composition in the Elderly

**DOI:** 10.3389/fmicb.2017.01757

**Published:** 2017-09-12

**Authors:** Hirosuke Sugahara, Shinsaku Okai, Toshitaka Odamaki, Chyn B. Wong, Kumiko Kato, Eri Mitsuyama, Jin-Zhong Xiao, Reiko Shinkura

**Affiliations:** ^1^Next Generation Science Institute, Morinaga Milk Industry Co., Ltd. Zama, Japan; ^2^Applied Immunology, Graduate School of Biological Science, Nara Institute of Science and Technology Ikoma, Japan

**Keywords:** gut microbiota, aging, IgA-seq, IgA, *Enterobacteriaceae*, *Clostridiaceae*

## Abstract

Gut microbiota is known to change with aging; however, the underlying mechanisms have not been well elucidated. Immunoglobulin A (IgA) is the dominant class of antibody secreted by the intestinal mucosa, and are thought to play a key role in the regulation of the gut microbiota. T cells regulate the magnitude and nature of microbiota-specific IgA responses. However, it is also known that T cells become senescent in elderly people. Therefore, we speculated that the age-related changes of IgA response against the gut microbiota might be one of the mechanisms causing the age-associated changes of gut microbiota composition. To prove our hypothesis, fecal samples from 40 healthy subjects (adult group: *n* = 20, an average of 35 years old; elderly group: *n* = 20, an average of 76 years old) were collected, and the gut microbiota composition and the response of IgA to gut microbiota were investigated. The relative abundance of *Bifidobacteriaceae* was significantly lower, whereas those of *Clostridiaceae*, *Clostridiales*;f__ and *Enterobacteriaceae* were significantly higher in the elderly group than in the adult group. There was no significant difference in the fecal IgA concentration between the adult and elderly groups. However, the taxon-specific IgA response to some bacterial taxa was different between the adult and elderly groups. To evaluate inter-group differences in the taxon-specific IgA response to each bacterial taxon, the IgA-indices were calculated, and the IgA-indices of *Clostridiaceae* and *Enterobacteriaceae* were found to be significantly lower in the elderly group than the adult group. In addition, *Clostridiales*;f__ and *Enterobacteriaceae* were significantly enriched in the IgA^+^ fraction in the adult group but not in the elderly group, whereas *Clostridiaceae* was significantly enriched in the IgA^-^ fraction in the elderly group but not in the adult group. Some species assigned to *Clostridiaceae* or *Enterobacteriaceae* are known to be pathogenic bacteria. Our results suggest the possible contribution of decreased IgA response in the increased abundance of bacterial taxa with potential pathogenicity in the intestinal environment of the elderly. Our findings contribute to the understanding of the regulatory factor for the changes in the gut microbiota composition with aging.

## Introduction

Human gut is inhabited by a trillion of microorganisms which constitute the gut microbiota (Clemente et al., 2012). Some diseases, such as inflammatory bowel disease, autoimmune and allergic diseases, obesity and diabetes, have been suggested as a result of dysbiosis induced by the changes in the types and numbers of gut microbiota (Clemente et al., 2012). The changes of gut microbiota are affected by lifestyle, diet, and xenometabolites (Marchesi et al., 2016). Furthermore, many reports have demonstrated that the gut microbiota composition changes during the aging process (Mitsuoka and Hayakawa, 1973; Mueller et al., 2006; Mariat et al., 2009; Mitsuoka, 2014; Arboleya et al., 2016; Odamaki et al., 2016). For instance, our previous study revealed that the relative abundance of Actinobacteria (*Bifidobacteriaceae*) substantially decreased after weaning and continued to decrease with age, whereas the relative abundance of Proteobacteria (*Enterobacteriaceae*) increased in subjects over 70 years old (Odamaki et al., 2016). However, the factors that regulate changes in the gut microbiota composition during aging are not well understood.

It is known that the gut mucosal immune response is significantly compromised in the elderly, and this decline with aging is associated with diminished antigen-specific antibody titre in the intestinal environment (Mabbott et al., 2015). Immunoglobulin A (IgA), which is the major class of antibody secreted by the gut mucosa, is thought to be one of the key factors for the maintenance of intestinal homeostasis (Suzuki et al., 2004; Peterson et al., 2007). IgA recognizes disease-driving inflammatory bacteria (e.g., *Bacteroides fragilis* and *Clostridium perfringens*) in humans wherein IgA-targeted elimination of such bacteria could potentially prevent disease development (Palm et al., 2014). Moreover, polyreactive IgA was reported to possess a strong impact on the generation of antigen-specific IgA and the selection and maintenance of the gut microbiota (Fransen et al., 2015). Okai et al. (2016) revealed that polyreactive monoclonal IgA with a high binding affinity to bacterial serine hydroxymethyltransferase identifies targeted microbes such as *Escherichia coli* and suppresses the growth of the microbes.

Previous reports showed that the age-associated changes of gut microbiota composition are related to changes of diet, gastrointestinal motility, mastication, and immunosenescence (Quercia et al., 2014). In particular, changes in systemic immune responses with aging affect immune function of T cells that regulate the magnitude and nature of microbiota-specific IgA responses (Mabbott et al., 2015). However, the association between the changes in gut microbiota composition during aging process and intestinal IgA response to the gut microbiota is not well understood. In this study, we demonstrated the association between the changes of gut microbiota composition with aging and taxon-specific IgA response.

## Materials and Methods

### Subjects

To evaluate the changes in the gut microbiota composition that occur with aging, 40 healthy subjects, who were selected from community-dwelling Japanese volunteers (one sample per subject), were assigned to two groups: adult group (*n* = 20, aged 33–39 years old with an average of 35 years old, male and female = 1:1) and elderly group (*n* = 20, aged 71–84 years old with an average of 76 years old, male and female = 1:1).

### Ethics Statement

All subjects were enrolled in the study protocol approved by the ethics committee of Kensyou-kai Incorporated Medical Institution (Osaka, Japan, approval No. 20170210-5). Written informed consent was obtained from all subjects. The consent forms signed by each participant included their consent to allow us to publish our findings.

### Sampling and Storage of Samples

Fecal sampling started in December 2014 and continued till May 2015. Briefly, fresh fecal samples were collected and transferred by the subjects into tubes and immediately enclosed in plastic bags containing AnaeroPouch (Mitsubishi Gas Chemical, Tokyo, Japan) to create an anaerobic environment. The fecal samples collected from subjects were stored at -20°C and transported to the laboratory by logistics companies.

### Sample Preparation

Sample preparation was performed as previously described (Palm et al., 2014) with some modifications. Human feces were placed in tubes containing 1.0 φ zirconia beads (ZB-10; TOMY, Tokyo, Japan) and incubated in 1 ml phosphate buffered saline (PBS) per 100 mg feces on ice for 1 h. Feces were homogenized by bead beating and then centrifuged (50 × *g*, 15 min, 4°C) to remove large particles. Fecal bacteria in the supernatants were placed in staining buffer, which is made of PBS containing 1% (w/v) bovine serum albumin (BSA; Wako Tokyo, Japan). After centrifugation (8000 × *g*, 5 min, 4°C), the supernatant was collected for measurement of IgA concentrations. The bacterial pellet was washed twice with staining buffer and the pellet was suspended in staining buffer containing 20% (v/v) normal mouse serum (Abcam Japan, Tokyo, Japan), incubated for 20 min on ice, and then stained with staining buffer containing 9.1% (v/v) PE-conjugated Anti-Human IgA (Clone IS11-8E10; Miltenyi Biotec, Bergisch Gladbach, Germany) for 30 min on ice. Sample was then washed three times with staining buffer and suspended in PBS containing 0.5% (w/v) BSA, 2 mM ethylenediaminetetraacetic acid (EDTA), and 4.7% (v/v) anti-PE magnetic activated cell sorting (MACS) beads (Miltenyi Biotec). After incubation for 15 min on ice, the sample was washed with PBS containing 0.5% (w/v) BSA and 2 mM EDTA (MACS buffer) and resuspended in MACS buffer.

The suspension was divided into three samples and details of the sample processing procedures are described in the Supplementary Information. The first sample was centrifuged (10000 × *g*, 5 min, 4°C) and used as a pre-sort fraction for 16S rRNA gene sequencing analysis. The second sample was used for the analysis of IgA-binding ability as the ratio of IgA-coated bacteria against the whole bacterial community by the fluorescence activated flow cytometry (SH800; Sony, Tokyo, Japan). The third sample was sorted by MACS (LS column; Miltenyi Biotec) into IgA-uncoated and IgA-coated bacteria. The IgA-uncoated bacteria were centrifuged (10000 × *g*, 5 min, 4°C) and the pellet was used as an IgA-uncoated fraction for 16S rRNA gene sequencing analysis. The IgA-coated bacteria were further purified via fluorescence activated cell sorter (SH800; Sony, Tokyo, Japan). The fraction of IgA-coated bacteria was pelleted (10000 × *g*, 5 min, 4°C) and used as an IgA-coated fraction for 16S rRNA gene sequencing analysis. All samples were stored at -80°C for future use.

### Microbiota Analysis

DNA was extracted from the fecal samples by the bead-beating method (Sugahara et al., 2015) and 16S rRNA gene sequencing was performed as previously described with minor modification (Odamaki et al., 2016). Briefly, the V3-V4 region of the bacterial 16S rRNA gene was amplified in triplicate by PCR using the TaKaRa Ex Taq HS Kit (TaKaRa Bio, Shiga, Japan) with the following program: preheating at 94°C for 3 min; 30 cycles of denaturation at 94°C for 30 s, annealing at 50°C for 30 s and extension at 72°C for 30 s; and a terminal extension at 72°C for 5 min. A 1 μl sample of the combined PCR products was amplified with the barcoded primers adapted for Illumina MiSeq sequencing. The amplification was performed according to the program as described above except that only eight cycles were performed. The products were purified and quantified by commercial kits according to the manufacturer’s protocol (Odamaki et al., 2016). Equal amounts of the amplicons were pooled and purified by the GeneRead Size Selection Kit (Qiagen, Valencia, CA, United States) according to the manufacturer’s protocol. The pooled libraries were sequenced by an Illumina MiSeq instrument and the MiSeq v3 Reagent Kit (Illumina Inc, San Diego, CA, United States).

After the acquisition of Illumina paired-end reads, the bowtie-2 program (Langmead and Salzberg, 2012) (ver. 2-2.2.4) was used to remove the reads mapped to PhiX 174 sequence and Genome Reference Consortium human build 37 (GRCh37). Thereafter, the 3′ region of each read with a PHRED quality score of less than 17 was trimmed. Trimmed reads less than 150 bp in length with an average quality score of less than 25 or those lacking paired reads were also removed. The trimmed paired-end reads were combined by the fastq-join script in EA-Utils (Aronesty, 2013) (ver. 1.1.2-537). Potential chimeric sequences were removed by reference-based chimera checking in USEARCH (Edgar et al., 2011) (ver. 5.2.32) and the gold database.^1^ The non-chimeric sequences (average ± standard deviation: 46171 ± 15242) were analyzed in the QIIME software package version 1.8.0 (Caporaso et al., 2010; Kuczynski et al., 2012). For analysis of family level, the sequences were assigned to operational taxonomic units (OTUs) by open-reference OTU picking (Rideout et al., 2014) with a 97% pairwise identity threshold and the Greengenes reference database (McDonald et al., 2012).^2^ All bacterial taxa were summarized at the family level.

### IgA Quantification of Fecal Samples

The amount of IgA in the supernatant of fecal suspension was quantified by ELISA kits (R&D systems, Minneapolis, MN, United States) in accordance with the manufacturer’s instructions.

### Calculation of Relative Abundances of Bacterial Taxa in IgA-Positive and -Negative Fractions

The calculation of the relative abundances of bacterial taxa in IgA fractions was performed in accordance to previous reports (Palm et al., 2014; Kau et al., 2015; Okai et al., 2016; Planer et al., 2016) with some modifications. It was noted that the previous studies did not take into consideration of the differences of the ratio of IgA-coated bacteria among individuals. To give a fair comparison, the relative abundances of bacterial taxa in IgA-positive (IgA^+^) and IgA-negative (IgA^-^) fractions, which have taken into consideration of the IgA-binding ability as the ratio of IgA-coated bacteria against the whole bacterial community, were used in this study. Relative abundances of bacterial taxa in IgA^+^ or IgA^-^ fractions were calculated based on the relative abundance of bacterial taxa in IgA-coated or IgA-uncoated fractions and the IgA-binding ability.

$${\text{IgA}}_{\text{taxon abundance}}^{+}=\text{IgA-coated}{\text{ fraction}}_{\text{taxon abundance}}\times \text{the IgA binding ability/100}$$

$${\text{IgA}}_{\text{taxon abundance}}^{-}=\text{IgA-uncoated }{\text{fraction}}_{\text{taxon abundance}}\times \left(1-\text{the IgA binding ability/100}\right)$$

### Calculation of IgA-Index

Immunoglobulin A-index was calculated based on the formula as described in the previous reports (Kau et al., 2015; Planer et al., 2016) with some modifications.

$$\text{IgA-index}=-\left[\text{log}({\text{IgA}}_{\text{taxon abundance}}^{+})-\text{log}\left({\text{IgA}}_{\text{taxon abundance}}^{-}\right)\right]/[\text{log}({\text{IgA}}_{\text{taxon abundance}}^{+})+\text{log}\left({\text{IgA}}_{\text{taxon abundance}}^{-}\right)]$$

The relative abundances of bacterial taxa in IgA^+^ or IgA^-^ fractions as described in the previous section were used. The IgA-index indicates the differential representation of a given taxon between the IgA^+^ and IgA^-^ fractions. The value of the IgA-index can range from a maximum of 1.0 (absent in IgA^-^_taxon abundance_) to a minimum of -1.0 (absent in IgA^+^_taxon abundance_).

### Statistical Analyses

Mann–Whitney *U* test and Wilcoxon signed-rank test were performed with SPSS version 22.0 statistical software (SPSS, Inc., Chicago, IL, United States). For all analyses, *p*-values of < 0.05 were considered statistically significant.

### Accession Numbers

DNA sequences of 16S rRNA gene metagenome data were deposited at DDBJ under accession numbers DRA005602.

## Results

### Differences in the Gut Microbiota Composition between Adults and the Elderly

**FIGURE 1:**
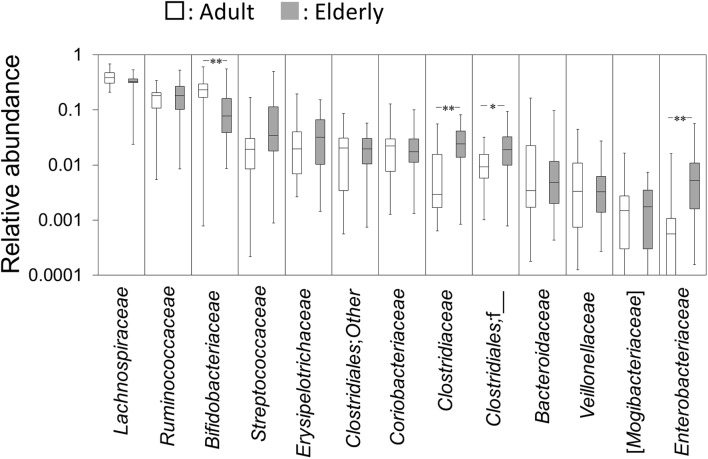
Abundances of bacterial taxa at family level. Gut microbiota composition in the adult and elderly groups are shown as boxes that denote the interquartile range between the first and third quartiles and the line within denotes the median (*n* = 20). The vertical axis is indicated by a logarithmic scale. *P*-values compared with the adult group were calculated using the Mann–Whitney *U* test. ^∗^*P* < 0.05; ^∗∗^*P* < 0.01.

The pre-sort fractions of the 40 healthy subjects were evaluated for differences in the gut microbiota composition between adults and the elderly. Taxon abundances with a median of more than 0.1 % at family level are shown in **Figure 1**. The relative abundance of *Bifidobacteriaceae* was significantly lower, whereas the relative abundances of *Clostridiaceae*, *Clostridiales*;f__, and *Enterobacteriaceae* were significantly higher in the elderly group compared to the adult group (**Figure 1**). No significant between-group difference was observed on other taxa.

### Differences in the Amount of Fecal IgA and the Ratio of IgA-Coated Bacteria to Gut Microbial Community between Adults and the Elderly

**FIGURE 2:**
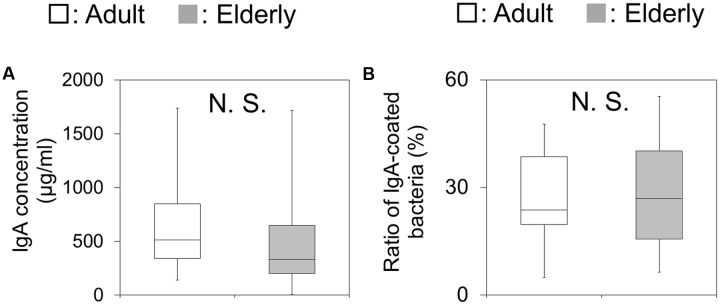
Fecal immunoglobulin A (IgA) concentrations and the ratio of IgA-coated bacteria in fecal samples. **(A)** The amounts of IgA in fecal samples are shown as boxes that denote the interquartile range between the first and third quartiles and the line within denotes the median (*n* = 20). *P*-values were calculated using the Mann–Whitney *U* test. N.S. indicates no significant difference. **(B)** The ratios of IgA-coated bacteria in fecal samples are shown as boxes that denote the interquartile range between the first and third quartiles and the line within denotes the median (*n* = 20). *P*-values were calculated using the Mann–Whitney *U* test. N.S. indicates no significant difference.

The IgA concentrations were not significantly different between the two groups (**Figure 2A**). In addition, the IgA-binding ability, which was measured by flow cytometric analysis as the ratio of IgA-coated bacteria against the total bacteria, showed no significant difference between the two groups (**Figure 2B**).

### Differences in the Taxon Abundances of IgA^+^ and IgA^-^ Fractions between Adults and the Elderly

The relative abundances of the bacterial taxa in IgA^+^ and IgA^-^ fractions against the whole microbial community were compared between the two groups. Similar to the analysis of pre-sort fractions, the relative abundance of *Bifidobacteriaceae* in IgA^-^ fractions was significantly lower, whereas the relative abundances of *Clostridiaceae*, *Clostridiales*;f__, and *Enterobacteriaceae* in IgA^-^ fractions were significantly higher in the elderly group compared with adult group (Supplementary Figure S2). However, in contrast to IgA^-^ fraction, the relative abundances of *Streptococcaceae* in IgA^+^ fractions was significantly higher in the elderly group compared with adult group (Supplementary Figure S3). These results indicate the possible changes with aging in the IgA response to some bacterial taxa.

### Differences in the Taxon-Specific IgA Response to the Gut Microbiota between Adults and the Elderly

**FIGURE 3:**
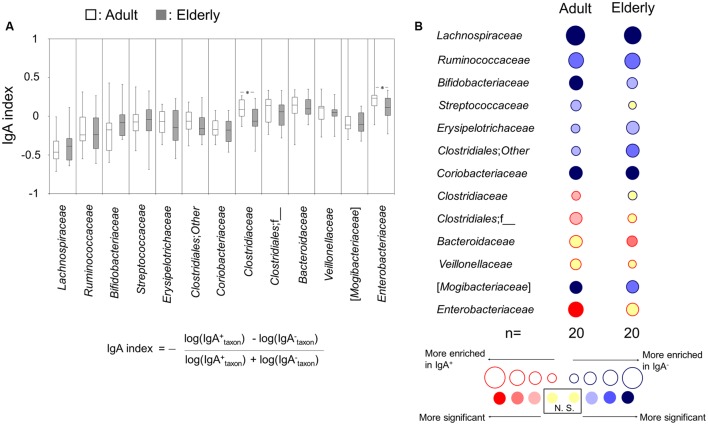
Immunoglobulin A-seq-based analysis for difference in taxon-specific IgA response between adults and the elderly. **(A)** IgA-indices of bacterial taxa are shown as boxes that denote the interquartile range between the first and third quartiles and the line within denotes the median (*n* = 20). *P*-values were calculated using the Mann–Whitney *U* test. ^∗^*P* < 0.05. Calculation formula for IgA-index is shown. **(B)** Bubble plots for taxon abundance show enrichment in either IgA^-^ or IgA^+^ fractions. The size of the circle and the color of the line indicate the magnitude of enrichment (mean IgA-index value) in either fraction. The internal color intensity indicates the statistical significance, as judged by a Wilcoxon signed-rank test. N.S. indicates no significant difference.

The IgA-indices, which were calculated for the evaluation of the inter-group differences of the taxon-specific IgA response to each bacterial taxon between the adult and elderly groups, of *Clostridiaceae* and *Enterobacteriaceae* in the adult group were significantly higher than those in the elderly group (**Figure 3A**). The taxon-specific IgA response was further analyzed by comparing the relative abundances of each taxon between IgA^+^ and IgA^-^ fractions by a Wilcoxon signed-rank test to determine whether the abundances were enriched in IgA^+^ or IgA^-^ fractions, for the adult and elderly groups, respectively. The relative abundances of *Clostridiaceae*, *Clostridiales*;f__, and *Enterobacteriaceae* were significantly enriched in the fraction of IgA^+^ in the adult group, but not in the elderly group (**Figure 3B**). The abundance of *Streptococcaceae* was significantly enriched in the fraction of IgA^-^ in the adult group, but not in the elderly group (**Figure 3B**). Meanwhile, the abundance of *Bacteroidaceae* was significantly enriched in the fraction of IgA^+^ in the elderly group, but not in the adult group (**Figure 3B**). There was no difference in the enrichment for other bacterial taxa (**Figure 3B**). These results indicate the difference of taxon-specific IgA response to some bacterial taxa between the adult and elderly groups.

## Discussion

The present study showed that the abundances of some bacterial groups in the gut microbiota were significantly different between adult and elderly groups whereby a lower abundance of *Bifidobacteriaceae* but a higher abundance of *Clostridiaceae*, *Clostridiales*;f__, and *Enterobacteriaceae* were observed in the elderly group than in the adult group. In agreement with the present study, previous studies indicated that the *Bifidobacteria* in human gut decreased with aging (Mitsuoka and Hayakawa, 1973; Mitsuoka, 2014; Arboleya et al., 2016; Odamaki et al., 2016) while *Enterobacteriaceae* and *C. perfringens* which is assigned to the family *Clostridiaceae* increased in elderly people (Mitsuoka and Hayakawa, 1973; Mariat et al., 2009; Mitsuoka, 2014; Odamaki et al., 2016).

*Bifidobacteria* belonging to *Bifidobacteriaceae* are believed to play a central role in maintaining a healthy intestinal environment (Bottacini et al., 2014). On the other hand, *Enterobacteriaceae* is thought to be the potential pathogenic microbes. Members of *Enterobacteriaceae* act in concert with the gut microbiota to induce colitis (Garrett et al., 2010) and are often associated with colorectal cancer (Bonnet et al., 2014). The relative abundance of *Enterobacteriaceae* is elevated in non-alcoholic steatohepatitis (Zhu et al., 2013) and an increased abundance of *Enterobacteriaceae* is used for exacerbation index of microbial dysbiosis (Gevers et al., 2014). In addition, some species in *Clostridiaceae* such as *Clostridium septicum*, *C. tetani*, and *C. perfringens* are known to be pathogenic (Samul et al., 2013). The characteristic of *Clostridiales*;f__ remains unclear since no bacterial species has been isolated from this family. The representative OTUs assigned to *Clostridiales*;f__ were closed to *Roseburia* sp. or *Eubacterium ramulus* (Supplementary Table S1). The genus *Roseburia* is the commensal microbes in human gut producing butyrate that affects colonic motility, immunity maintenance, and anti-inflammatory properties (Tamanai-Shacoori et al., 2017). *Eubacterium ramulus* is the commensal microbes in human gut that is able to degrade flavonol quercetin and the flavone luteolin (Braune et al., 2001). Although the fundamental characteristics of *Clostridiales*;f__ remain unclear; bacterial species assigned to this family are possibly non-pathogenic microbes. Taken together, with the exception of *Clostridiales*;f__, the gut microbiota compositional differences observed in this study illustrated the decrease of beneficial microbes and increase of the potential pathogenic microbes in abundance in human gut with aging.

Consistent with previous study which showed that the IgA concentration in intestinal environment was not significantly different between adult and elderly groups (Arranz et al., 1992), our result showed that there was no significant difference in the fecal IgA concentration between the adult and elderly groups. These results indicate that the amount of IgA did not change with aging. Consistent with previous study which showed that the gut microbiota was highly coated by IgA (van der Waaij et al., 1994, 1996), all bacterial taxa were detected in the IgA^+^ fractions. Previous studies indicated that patients with inflammatory bowel disease had an increased ratio of IgA-coated bacteria (van der Waaij et al., 2004; Palm et al., 2014). However, in the present study, the ratio of IgA-coated gut microbiota against the whole bacterial community was not significantly different between the adult and the elderly groups. These results suggest that the IgA-binding ability in the healthy population did not change with aging.

However, we found the potential differences in taxon-specific IgA response. Several bacterial taxa (*Bifidobacteriaceae, Clostridiaceae, Clostridiales*;f__, and *Enterobacteriaceae*) displayed significant differences in the comparisons of both the pre-sort and IgA^-^ fractions but not the IgA^+^ fractions, and *Streptococcaceae* displayed significant difference in the comparison of IgA^+^ fraction but not both of the pre-sort and IgA^-^ fractions. These results suggest the possibility of difference in IgA response to some bacterial taxa between the adult and elderly groups. Hence, to evaluate the differences in the taxon-specific IgA response between adults and the elderly, we calculated the IgA-index and determined the enrichment of bacterial taxa by the comparison between the taxon abundances in IgA^-^ and IgA^+^ fractions of each group. The IgA-indices of *Clostridiaceae* and *Enterobacteriaceae* were significantly lower in the elderly group compared with the adult group. Furthermore, the enrichment of *Streptococcaceae*, *Clostridiaceae, Clostridiales*;f__, *Bacteroidaceae*, and *Enterobacteriaceae* was different between the two groups. These results indicated that taxon-specific IgA response to *Clostridiaceae* and *Enterobacteriaceae* decreased with aging. However, there was no difference for the IgA index as well as the enrichment of *Bifidobacteriaceae* between the two groups.

Considered together, the findings in our present study demonstrate an association between the decreased taxon-specific IgA response and the increased abundance of *Clostridiaceae* and *Enterobacteriaceae*, which are known to be the bacterial groups with potential pathogenicity. Although there might be other unknown mechanisms that affecting the changes in gut microbiota composition with aging, our data suggest a causative effect of the decreased taxon-specific IgA response in the increased abundance of these bacteria in the elderly. It is known that antigen-specific IgA and high-affinity IgA are important for targeted elimination of undesirable microbes (Fransen et al., 2015; Okai et al., 2016), however, such antigen-specific IgA possess a lower affinity in the elderly (Mabbott et al., 2015). The reduction of intestinal antigen-specific IgA responses is known to be associated with aging (Fujihashi et al., 2000; Fujihashi and Kiyono, 2009). In addition, it was reported that the taxon-specific IgA response to certain bacterial taxa was responsible for regulating the abundance of that bacterial taxa in mice (Mirpuri et al., 2014).

Furthermore, it is known that T cells regulate the magnitude and nature of microbiota-specific IgA responses, thereby affecting the composition of gut microbiota (Honda, 2015). For instance, T cells promote antigen-specific IgA response via differentiation to T follicular helper cells (Honda, 2015). Since T cells immunosenescence is occurred in elderly people under a steady-state condition (Isobe et al., 2017), the decreased IgA response against bacterial groups with potential pathogenicity as demonstrated in our study may be attributed to T cell immunosenescence that occurs with aging. However, further analysis is needed to understand the regulating mechanisms of IgA response in the intestinal environment.

In addition, recent review predicted that IgA support a symbiosis of beneficial microbes in the intestinal environment (Kubinak and Round, 2016). Indeed, a monoclonal intestinal IgA with high-affinity against colitogenic bacteria such as *Escherichia coli* did not suppress the growth of beneficial bacteria such as *Lactobacillus casei* (Okai et al., 2016). Consistent with the previous report, our findings revealed that the taxon-specific IgA response to *Bifidobacteriaceae* and *Clostridiales*;f__ did not show significant differences between the adults and elderly groups, indicating that IgA may not suppress the beneficial and non-pathogenic microbes. However, further analysis is needed to understand the mechanisms for the compositional changes of beneficial and non-pathogenic microbes that occur with aging.

## Conclusion

We demonstrated the association of decreased IgA response and the increased abundance of bacterial taxa with potential pathogenicity in the elderly as compared with the adult groups. This is the first report about the reduction of taxon-specific IgA response with aging in a human study. The reduction of taxon-specific IgA response may be one of the mechanisms for the compositional changes of gut microbiota in the elderly. We believe that our findings are valuable for understanding the regulatory mechanisms of the gut microbiota compositional change during the aging process.

## Author Contributions

HS, TO, J-ZX, and RS conceived and designed the experiments. HS, SO, KK, and EM performed the experiments. HS analyzed the data. SO provided analytical tools. HS, TO, CW, J-ZX, and RS wrote the paper.

## Conflict of Interest Statement

HS, TO, CW, KK, EM, and J-ZX are employees of Morinaga Milk Industry Co., Ltd. The other authors declare that the research was conducted in the absence of any commercial or financial relationships that could be construed as a potential conflict of interest.

## Footnotes

1 http://drive5.com/otupipe/gold.tz

2 ftp://greengenes.microbio.me/greengenes_release/gg_13_5/gg_13_8_otus.tar.gz
