# Supplementary material for: Decreased Taxon-Specific IgA Response in Relation to the Changes of Gut Microbiota Composition in the Elderly
Source: Front Microbiol. 2017 Sep 12;8:1757. doi: 10.3389/fmicb.2017.01757 (PMC5601059; doi:10.3389/fmicb.2017.01757)
Supplement: Supplementary file 1 [file Presentation_1.PDF]

# - Supplementary Material -

Decreased taxon-specific IgA response in relation to the changes of gut microbiota composition in the elderly

Hirosuke Sugahara[1], Shinsaku Okai[2], Toshitaka Odamaki[1], Chyn Boon Wong[1], Kumiko Kato[1], Eri Mitsuyama[1], Jin-zhong Xiao[1]* and Reiko Shinkura[2]

1 Next Generation Science Institute, Morinaga Milk Industry Co., Ltd., 1-83, 5-Chome, Higashihara, Zama-City, Kanagawa, Japan
2 Applied Immunology, Graduate School of Biological Science, Nara Institute of Science and Technology, 8916-5, Takayama-cho, Ikoma-City, Nara, Japan

Supplementary methods

Confirmation of separation method of IgA-uncoated and IgA-coated bacteria

Sample preparation described in this section was performed as previously described (Palm et al., 2014) with some modifications. Human faeces were placed in tubes containing 1.0 φ zirconia beads (ZB-10; TOMY, Tokyo, Japan) and incubated in 1 ml phosphate buffered saline (PBS) per 100 mg faeces on ice for 1 h. Faeces were homogenised by bead beating and then centrifuged (50 g, 15 min, 4°C) to remove large particles. Faecal bacteria in the supernatants were placed in staining buffer, which is made of PBS containing 1 % (w/v) bovine serum albumin (BSA; Wako Tokyo, Japan). The bacterial pellet was washed twice with staining buffer and the pellet was suspended in staining buffer containing 20% (v/v) normal mouse serum (Abcam Japan, Tokyo, Japan), incubated for 20 min on ice. For confirmation of separation method, the sample was divided to three samples.

For deciding on IgA-coated fraction, the first sample treated with mouse serum was washed 3 times with staining buffer and suspended in PBS containing 0.5% (w/v) BSA, 2mM ethylenediaminetetraacetic acid (EDTA), and 4.7% (v/v) Anti-PE Magnetic Activated Cell Sorting (MACS) beads (Miltenyi Biotec). After incubation for 15 min on ice, the sample was washed with PBS containing 0.5% (w/v) BSA and 2 mM EDTA

(MACS buffer) and resuspended in MACS buffer. Thereafter, the sample was analysed by fluorescence activated flow cytometry (SH800; Sony, Tokyo, Japan) to decide on the IgA-coated fraction (Non-stained sample; Supplementary FigureS1 A).

For analysing IgA-coated bacteria, the second sample treated with mouse serum was stained with staining buffer containing 9.1% (v/v) PE-conjugated Anti-Human IgA for 30 min on ice. Sample was then washed 3 times with staining buffer and suspended in PBS containing 0.5% (w/v) BSA, 2mM EDTA, and 4.7% (v/v) Anti-PE MACS beads. After incubation for 15 min on ice, the sample was washed with MACS buffer and resuspended in MACS buffer. Thereafter, the sample was analysed by fluorescence activated flow cytometry to analyse the IgA-coated bacteria (Pre-sort sample; Supplementary FigureS1 B).

For confirming enrichment in IgA-uncoated and -coated bacteria, the third sample was sorted by MACS (LS column; Miltenyi Biotec) into IgA-uncoated and IgA-coated bacteria. IgA-uncoated bacteria was analysed by fluorescence activated flow cytometry in order to confirm enrichment of the IgA-uncoated bacteria (IgA-uncoated bacteria; Supplementary FigureS1 C). The IgA-coated bacteria were further purified via fluorescence activated cell sorter. The purified IgA-coated bacteria was analysed by the fluorescence activated flow cytometry to confirm enrichment of the IgA-coated bacteria

(IgA-coated bacteria; Supplementary FigureS1 D).

Prediction of bacterial species assigned to *Clostridiales*;f__

Representative OTUs, which were assigned to *Clostridiales*;f__ and were dominant in dataset (more than 5000 read in dataset of the present study), were predicted by a blastn program (stand-alone BLAST version 2.2.28+) with an 99 % similarity. A 16S database described in DDBJ database (published in Dec 2016) was used as a reference database.

Supplementary reference

Palm, N. W., de Zoete, M. R., Cullen, T. W., Barry, N. A., Stefanowski, J., Hao, L., et al. (2014). Immunoglobulin A coating identifies colitogenic bacteria in inflammatory bowel disease. *Cell* 158, 1000–10. doi:10.1016/j.cell.2014.08.006.

Supplementary Figures

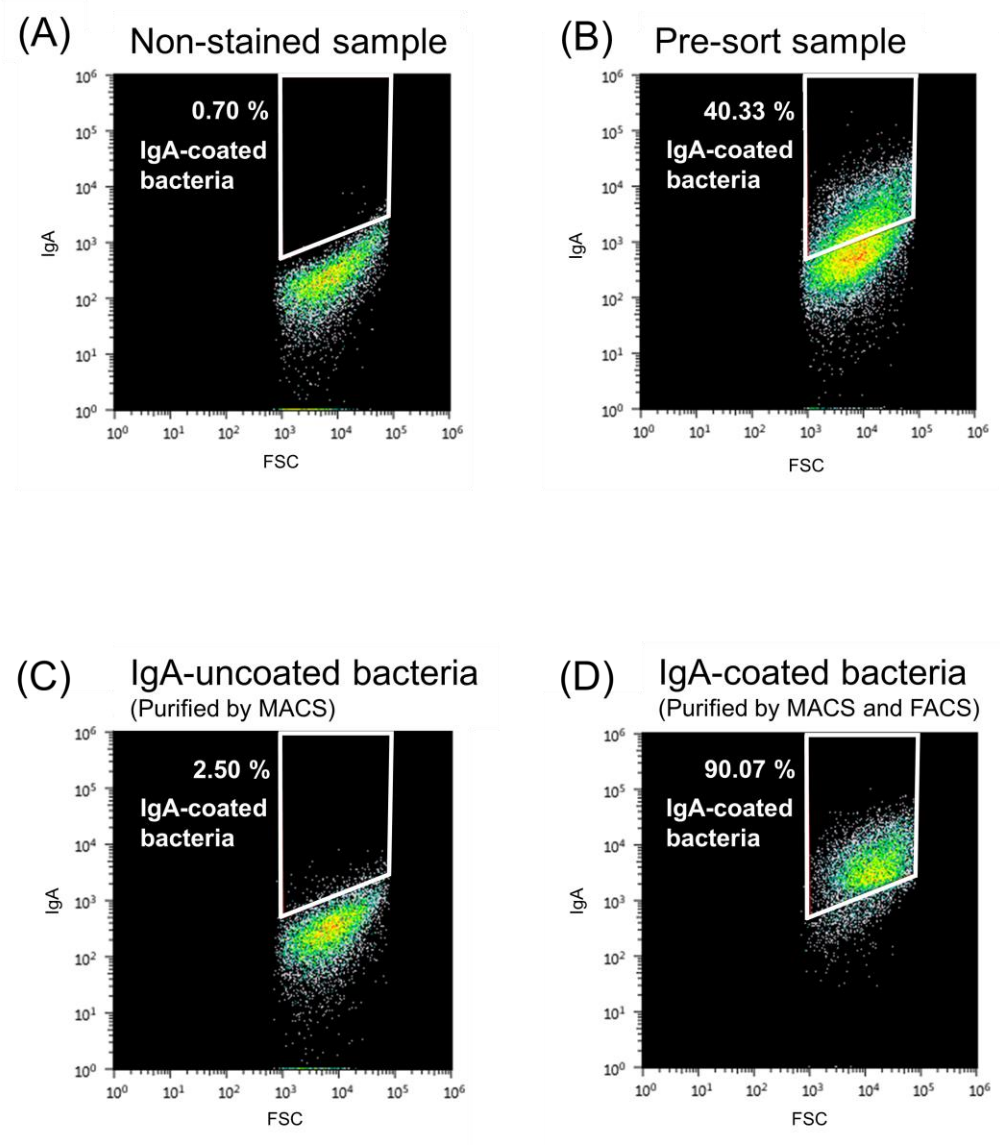

Supplementary Figure S1. Flow cytometric analysis of samples.

(A) The bacterial cell sorting result of sample without staining by PE-conjugated Anti-Human IgA. Representative fluorescence activated bacterial cell sorting results of **(B)** pre-sort, **(C)** IgA-uncoated and **(D)** IgA-coated bacteria. FACS indicates Fluorescence Activated Cell Sorting. FSC indicates forward scatter.

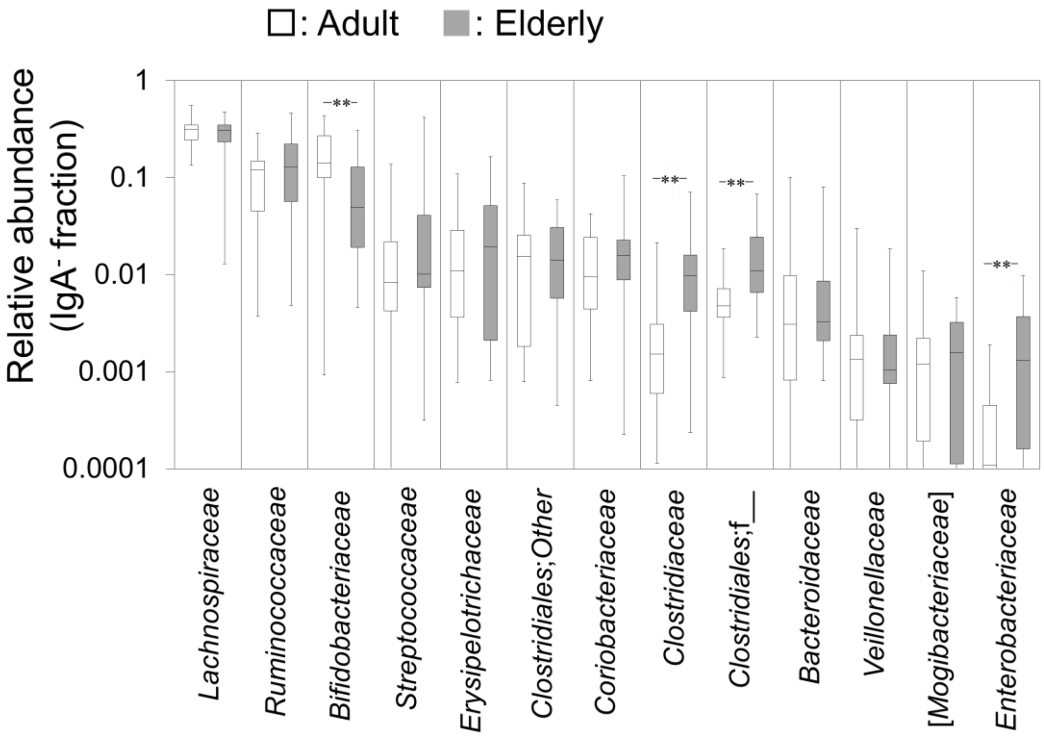

Supplementary Figure S2. Relative abundances of bacterial taxa in IgA$^-$ fraction

Taxon abundances in the IgA$^-$ fractions, which indicate the abundances against the whole microbial community, are shown as boxes that denote the interquartile range between the first and third quartiles and the line within denotes the median (n = 20). The vertical axis is indicated by a logarithmic scale. P-values were calculated using the Mann–Whitney U test. **P < 0.01.

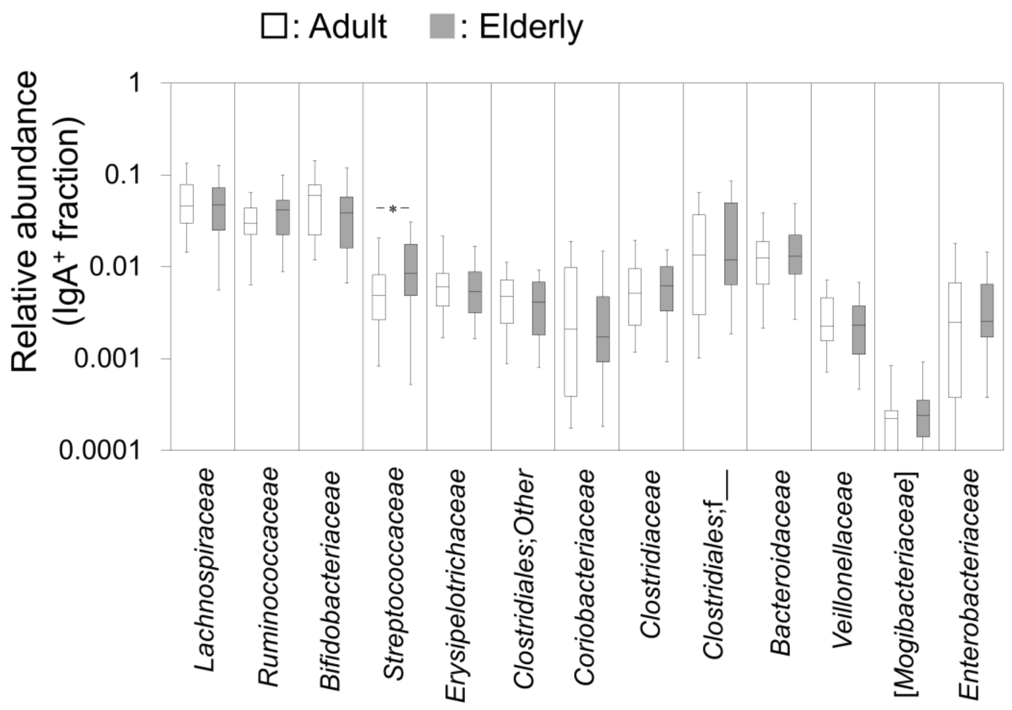

Supplementary Figure S3. Relative abundances of bacterial taxa in IgA^{+} fraction

Taxon abundances in IgA^{+} fractions, which indicate the abundances against the whole microbial community, are shown as boxes that denote the interquartile range between the first and third quartiles and the line within denotes the median (n = 20). The vertical axis is indicated by a logarithmic scale. P-values were calculated using the Mann–Whitney U test. **P < 0.05.

Supplementary Table

Supplementary Table S1. Prediction of bacterial species assigned to *Clostridiales*;f__

Representative OTUs	Sequence	Blast assignment (more than 99 % similarity)
Representative OTU1	GATATTGGACAATGGGGGGAACCCTGATCCAGCGACGCCGCGTGAGTGAAGAAGTATTTCGGTATGTAAAGCTCTATCAGCAGGGAAGAAGAAATGACGGTACCTGAGTAAGAAGCCCCGGCTAACTACGTGCCAGCAGCCGCGGTAATACGTAGGGGGCAAGCGTTATCCGGAATTACTGGGTGTAAAGGGAGCGTAGACGGTAAAGCAAGTCTGGAGTGAAAGGCTGGGGCCCAACCCCGGGACTGCTCTGGAAACTGTTAAACTGGAGTGCAGGAGAGGCAGGCGGAATTCCTAGTGTAGCGGTGAAATGCGTAGATATTAGGAGGAACACCAGTGGCGAAGGCGGCCTGCTGGACTGTAACTGACGTTGAGGCTCGAAAGCGTGGGGAGCAAACAGG	No hit
Representative OTU2	AATATTGCACAATGGGGGAAACCCTGATGCAGCGACGCCGCGTGGGTGAAGAAGTGATTCGTCACGTAAAGCCCTATCAGCAGGGAAGAAAATGACGGTACCTGAGTAAGAAGCCCCGGCTAACTACGTGCCAGCAGCCGCGGTAATACGTAGGGGGCAAGCGTTATCCGGATTTACTGGGTGTAAAGGGAGCGTAGACGGTGCGCCAAGTCTGGAGTGAAATGCCGCGGCTTAACCGCGGAACTGCTTTGGAAACTGGCGGGCTTGAGTGCGGGAGGGGTAAGCGGAATTCCTAGTGTAGCGGTGAAATGCGTAGATATTAGGAGGAACACCGGTGGCGAAGGCGGCTTACTGGACCGTAACTGACGTTGAGGCTCGAAAGCGTGGGGAGCAAACAGG	No hit
Representative OTU3	AATATTGCACAATGGGGGAAACCCTGATGCAGCGACGCCGCGTGAGCGATGAAGTATTTCGGTATGTAAAGCTCTATCAGCAGGGAAGATAATGACGGTACCTGACTAAGAAGCTCCGGCTAAATACGTGCCAGCAGCCGCGGTAATACGTATGGAGCAAGCGTTATCCGGATTTACTGGGTGTAAAGGGAGCGTAGGCGGTCCTGCAAGTCTGATGTGAAAACCCGGGGCTCAACCCCGGGACTGCATTGGAAACTGTAGGACTAGAGTGTCGGAGGGGTAAGTGGAATTCCTAGTGTAGCGGTGAAATGCGTAGATATTAGGAGGAACACCAGTGGCGAAGGCGGCTTACTGGACGACCACTGACGCTGAGGCTCGAAAGCGTGGGGAGCAAACAGG	*Eubacterium ramlus*
Representative OTU4	AATATTGCACAATGGGGGAAACCCTGATGCAGCGACGCCGCGTGAGTGAAGAAGTATTTCGGTATGTAAAGCTCTATCAGCAGGGAAGAAAGTGACGGTACCTGAATAAGAAGCCCCGGCTAACTACGTGCCAGCAGCCGCGGTAATACGTAGGGGGCAAGCGTTATCCGGATTTACTGGGTGTAAAGGGAGCGTAGACGGCAAGGCAAGTCTGAAGTGAAAGCCCGGTGCTTAACGCCGGGACTGCTTTGGAAACTGTTTGGCTGGAGTGCCGGAGAGGTAAGCGGAATTCCTAGTGTAGCGGTGAAATGCGTAGATATTAGGAAGAACACCAGTGGCGAAGGCGGCTTACTGGACGGTAACTGACGTTGAGGCTCGAAAGCGTGGGGAGCAAACAGG	No hit
Representative OTU5	AATATTGGGCAATGGGGGAAACCCTGACCCAGCAACGCCGCGTGAAGGAAGAAGGCCTTCGGGTTGTAAACTTCTTTTACCAGGGACGAAGGACGTGACGGTACCTGGAGAAAAAGCAACGGCTAACTACGTGCCAGCAGCCGCGGTAATACGTAGGTTGCAAGCGTTGTCCGGATTTACTGGGTGTAAAGGGCGTGTAGGCGGAGGCGCAAGTTGGGAGTGAAATCTATGGGCTCAACCCATAAACTGCTCTCAAAACTGTGCCCCTTGAGTATCGGAGAGGCAAGCGGAATTCCTAGTGTAGCGGTGAAATGCGTAGATATTAGGAGGAACACCAGTGGCGAAGGCGGCTTGCTGGACGACAACTGACGCTGAGGCGCGAAAGCGTGGGGAGCAAACAGG	No hit
Representative OTU6	AATATTGCACAATGGGGGAAACCCTGATGCAGCGACGCCGCGTGAGCGATGAAGTATTTCGGTATGTAAAGCTCTATCAGCAGGGAAGATAATGACGGTACCTGACTAAGAAGCTCCGGCTAAATACGTGCCAGCAGCCGCGGTAATACGTATGGAGCAAGCGTTATCCGGATTTACTGGGTGTAAAGGGAGCGTAGGCGGTCCTGCAAGTCTGATGTGAAAGGCCGGGGCTCAACCCCGGGACTGCATTGGAAACTGTAGGACTAGAGTGTCGGAGGGGTAAGTGGAATTCCTAGTGTAGCGGTGAAATGCGTAGATATTAGGAGGAACACCAGTGGCGAAGGCGGCTTACTGGACGACTACTGACGCTGAGGCTCGAAAGCGTGGGGAGCAAACAGG	*Eubacterium ramlus*
Representative OTU7	AATATTGCGCAATGGAGGAAACTCTGACGCAGTGACGCCGCGTATAGGAAGAAGGTTTTCGGATTGTAAACTATTGTCGTTAGGGAAGATAAAAGACTGTACCTAAGGAGGAAGCCCCGGCTAACTATGTGCCAGCAGCCGCGGTAATACATAGGGGGCAAGCGTTATCCGGAATTATTGGGTGTAAAGGGTGCGTAGACGGAGAAACAAGTTGGTTGTGAAATCCCTCGGCTCAACTGAGGAACTGCAACCAAAACTATTTCCCTTGAGTGTCGGAGAGGAAAGTGGAATTCCTAGTGTAGCGGTGAAATGCGTAGATATTAGGAGGAACACCAGTGGCGAAGGCGACTTTCTGGACGATAACTGACGTTGAGGCACGAAAGTGTGGGGAGCAAACAGG	No hit
Representative OTU8	AATATTGCACAATGGAGGAAACTCTGATGCAGCGACGCCGCGTGAGTGAAGAAGTATTTCGGTATGTAAAGCTCTATCAGCAGGGAAGAAAATGACGGTACCTGACTAAGAAGCACCGGCTAAATACGTGCCAGCAGCCGCGGTAATACGTATGGTGCAAGCGTTATCCGGATTTACTGGGTGTAAAGGGAGCGTAGGTGGCAAGGCAAGCCAGAAGTGAAAACCCGGGGCTCAACCGCGGGATTGCTTTTGGAACTGTCATGCTAGAGTGCAGGAGGGGTGAGCGGAATTCCTAGTGTAGCGGTGAAATGCGTAGATATTAGGAGGAACACCGGAGGCGAAGGCGGCTCACTGGACTGTAACTGACACTGAGGCTCGAAAGCGTGGGGAGCAAACAGG	*Roseburia* sp.
Representative OTU9	AATATTGCGCAATGGAGGAAACTCTGACGCAGTGACGCCGCGTATAGGAAGAAGGTTTTCGGATTGTAAACTATTGTCGTTAGGGAAGAAATTGACAGTACCTAAGGAGGAAGCTCCGGCTAACTATGTGCCAGCAGCCGCGGTAATACATAGGGAGCAAGCGTTATCCGGAATTATTGGGTGTAAAGGGTGCGTAGACGGGAAATTAAGTTAGTTGTGAAATCCCTTGGCTTAACTGAGGAACTGCAACTAAAACTGGTTTTCTTGAGTATTGGAGAGGAAAGTGGAATTCCTAGTGTAGCGGTGAAATGCGTAGATATTAGGAGGAACACCAGTGGCGAAGGCGACTTTCTGGACAATAACTGACGTTGAGGCACGAAAGTGTGGGGAGCAAACAGG	No hit
